# Glycosidically-Bound Volatile Phenols Linked to Smoke Taint: Stability during Fermentation with Different Yeasts and in Finished Wine

**DOI:** 10.3390/molecules26154519

**Published:** 2021-07-27

**Authors:** Brandon A. Whitmore, Stephanie E. McCann, Matthew Noestheden, Eric G. Dennis, Sarah M. Lyons, Daniel M. Durall, Wesley F. Zandberg

**Affiliations:** 1 Department of Biology, The University of British Columbia, 1177 Research Road, Kelowna, BC V1V 1V7, Canada; brandon.whitmore1@ucalgary.ca (B.A.W.); semccann@hotmail.ca (S.E.M.); sarah@suprarnd.ca (S.M.L.); daniel.durall@ubc.ca (D.M.D.); 2 Department of Chemistry, The University of British Columbia, 3247 University Way, Kelowna, BC V1V 1V7, Canada; noesmatt@gmail.com (M.N.); eric.dennis@ubc.ca (E.G.D.)

**Keywords:** smoke taint, volatile phenols, guaiacol, glycosides, yeast strains, fermentation, glycosidase, hydrolysis, aging

## Abstract

When wine grapes are exposed to smoke, there is a risk that the resulting wines may possess smoky, ashy, or burnt aromas, a wine flaw known as smoke taint. Smoke taint occurs when the volatile phenols (VPs) largely responsible for the aroma of smoke are transformed in grape into a range of glycosides that are imperceptible by smell. The majority of VP-glycosides described to date are disaccharides possessing a reducing β-d-glucopyranosyl moiety. Here, a two-part experiment was performed to (1) assess the stability of 11 synthesized VP-glycosides towards general acid-catalyzed hydrolysis during aging, and (2) to examine whether yeast strains differed in their capacity to produce free VPs both from these model glycosides as well as from grapes that had been deliberately exposed to smoke. When fortified into both model and real wine matrices at 200 ng/g, all VP-disaccharides were stable over 12 weeks, while (42–50 ng/g) increases in free 4-ethylphenol and *p*-cresol were detected when these were added to wine as their monoglucosides. Guaiacol and phenol were the most abundantly produced VPs during fermentation, whether originating from natural VP-precursors in smoked-exposed Pinot Noir must, or due to fortification with synthetic VP-glycosides. Significant yeast strain-specific differences in glycolytic activities were observed for phenyl-β-d-glycopyranoside, with two strains (RC212 and BM45) being unable to hydrolyze this model VP, albeit both were active on the guaiacyl analogue. Thus, differences in *Saccharomyces cerevisiae* β-glucosidase activity appear to be influenced by the VP moiety.

## 1. Introduction

Smoke taint is an increasingly common wine fault that has, and will likely continue to have, serious economic consequences for the global wine industry, particularly in fire-prone grape-growing and wine-producing regions. Smoke produced from the combustion of lignocellulose during prescribed burns or forest fires contains a range of volatile phenols (VPs) such as guaiacol, *o*-, *m*-, and *p*-cresol, 4-methylguaiacol, and syringol [1] that are largely responsible for its characteristic “burnt” odor [2]. Most of these VPs have low (part-per-billion) aroma thresholds, leading to their adoption as primary markers of smoke taint in both smoke-exposed grapes and the resulting wines [3]. Smoke-derived VPs may be absorbed into ripening grape tissues, where the majority are linked chemically to glucose by endogenous grape glycosyltransferases [4,5], a process which is thought to mitigate the cytotoxic effects of VPs [6]. The resultant VP-β-d-glucosides [7] may be further elongated to di- or trisaccharides, with the disaccharides representing the bulk of the in-grape VP-glycosides [8]. VP-glycosides, in contrast to their aglycones (i.e., free VPs), are non-volatile and hence do not possess the smoky or ashy aromas or flavors, albeit they may produce unpleasant retro-nasal aftertaste upon their in-mouth hydrolysis by oral bacteria [9]. Problematically, increases in free, perceptible (by smell and taste) VPs are frequently observed after fermenting juice or must derived from smoke-exposed grapes [3,10,11,12,13], resulting in the production of smoke-tainted wines even when the grapes did not initially possess objectionable smoky aromas. Like other glycosidically-bound aroma precursors, VP-glycosides are thought to be hydrolyzed either by microbial glycosidases [14] or via non-specific, general acid-catalyzed reactions [15] enabled by the low pH achieved during fermentation. Given that a substantial fraction of VP-glycosides survive enzyme-catalyzed hydrolysis during fermentation [8,10], the general acid hydrolysis mechanism is more problematic for wines that are aged.

In addition to research into improving VP detection methods and smoke taint risk assessments [10,11], studies on the amelioration or prevention of smoky aromas in wine and berries are of significant, practical importance to grape growers and winemakers [16,17]. In practice, wines tainted with unwanted phenolic compounds can be treated with fining agents which may physically adsorb or chemically complex with VPs in wine to facilitate their removal [17]. Activated carbon has been found to reduce 4-ethylphenol and 4-ethylguaiacol in red wines, while egg albumin, isinglass, chitosan, and carboxymethylcellulose were suggested as other potential agents [18]. Purified grape pomace has been shown to be more effective than other fining agents at removing 4-methylguaiacol from Monastrell wine [19], albeit the researchers did not investigate wine specifically tainted by smoke. A study of smoke-affected Pinot Noir wine found that activated carbon reduced the concentration of guaiacol, 4-methylguaiacol and cresols, while other treatments, such as isinglass and egg albumin, had no effect [20]. Alternatively, reverse osmosis and solid phase adsorption treatment of Pinot Noir tainted by straw-derived smoke reduced the concentration of VPs as well as perceived smoke aromas [17]. Fining or other methods of removing smoke compounds from wine may lower their concentration in the treated samples, but they typically encounter two confounding issues. First, these methods may not be selective enough and can remove desirable aroma or color compounds along with the unwanted VPs [20]. Second, removal of free VPs still leaves behind the glycosidically-bound forms which may be released as the wine ages (*vide infra*). After the reverse osmosis treatment, it has been observed that the taint was slowly returning, possibly due to hydrolysis of glycoconjugates left in wine [17], although subsequent researchers have demonstrated that VP-glycosides are quite stable at wine pH [8,21]. These latter studies would suggest that instead of treating the final product it would be more beneficial to prevent VP-glycoside hydrolysis during the initial wine making stages (or removing VP-glycosides prior to fermentation).

Several studies have investigated the effect of changing wine-making practices on both VP concentration and the subsequent perception of smoke taint. For example, duration of skin contact in red wine making significantly affects the levels of VPs released [22], as does crushing of the grapes before the pressing in white wine [16]. Other techniques that do not directly impact the levels of VPs should also be considered due to the effects on the overall aroma profile. Fermentation temperature, while having no influence on the concentration of guaiacol, 4-methylguaiacol, or 4-ethylguaiacol, did change the final aroma composition of Petit Verdot red wines, suggesting that a balance that shifts the sensory perception to more pleasant aromas may be achieved with changing temperature [23]. Similarly, different yeast strains, as well as addition of oak chips or tannins, can increase the complexity of aromas and flavors that mask the smoke taint in finished wine [22]. Postharvest ozone fumigation, although initially proposed for microbial control, has coincidentally been demonstrated to increase the “fruity” character of resulting wine, indicating possible changes in aroma compounds [24]. A recent study used ozone fumigation on harvested grapes after smoke application and reported that this treatment significantly reduced the concentrations of free guaiacol and 4-methylguaiacol [25]. However, a follow up study demonstrated that ozone only minimally affected the levels of VP-glycoconjugates in wines made from smoke-exposed grapes [26]. Understanding how winemaking techniques affect the volatile composition of the final product will aid in mitigating the perception of smoke taint. However, the risk of free VPs being released into the wine during aging remains unless the smoke aroma compounds in both free and bound forms are completely removed, or the rates of VP-glycoside hydrolysis are unambiguously established.

**Figure 1:**
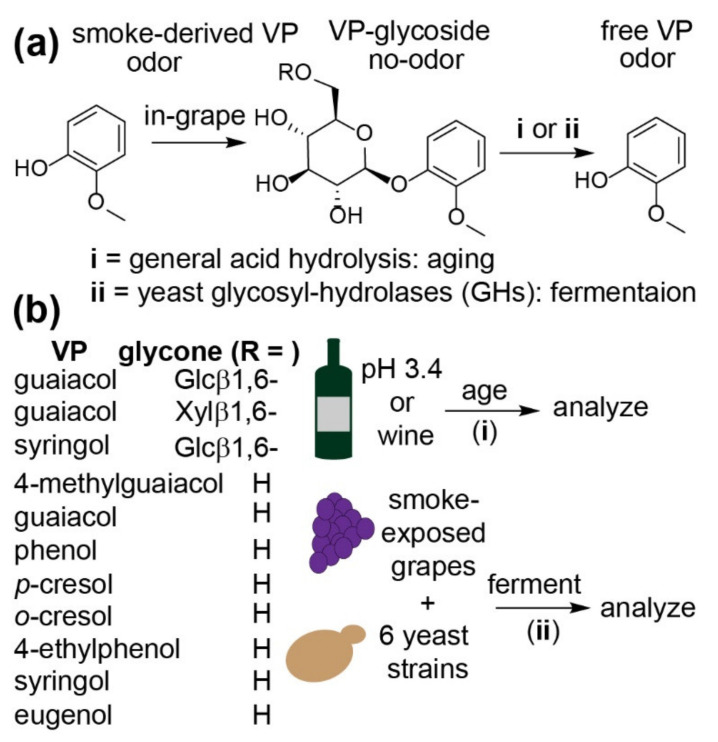
(**a**) Smoke-derived VPs are stored within ripening grape tissues as mono-β-d-glucosides (R = H) or a range of larger disaccharides (R = β-xylose, β-glycose, α-rhamnose, etc.), none of which possess the characteristic smoky odor of the free VPs. The hydrolysis of these VP-glycosides may be catalyzed either by the acidic pH of wine (i) or by the activity of yeast/bacterial glycosyl-hydrolases (ii), resulting in increased concentrations of free VPs during aging or primary fermentation, respectively. (**b**) Three VP-disaccharides (R = glucose (Glc) or xylose (Xyl)) and eight VP-mono-β-d-glucosides (R = H) were added to model and real wines to assess their stability towards hydrolysis; additionally, the same suite of model VP-glycosides was added to smoke-exposed grapes and matched controls to evaluate the ability of differing commercial yeast strains to affect their hydrolysis during primary fermentation.

One approach towards mitigating the effects of smoke taint that remains to be systematically explored is the use of fermentation conditions that prevent the hydrolysis of VP-glycosides. As noted, the rate of VP-glycoside hydrolysis is accelerated either via general acid or enzyme catalysis (routes i and ii, respectively, of Figure 1a). The majority of grape aroma glycosides are thought to be disaccharides composed of a reducing β-d-glucopyranoside and a non-reducing (i.e., the R group in Figure 1a) 6-*O*-β-d-apiofuranoside, 6-*O*-α-L-rhamnopyranoside, 6-*O*-β-d-glucopyranoside, or 6-*O*-α-L-arabinofuranoside moieties [6,7]. Therefore, the enzymatic hydrolysis of most of these VP-glycosides is considered to be a sequential two-step process involving first the hydrolysis of the non-reducing residue (e.g., by an α-rhamnosidase or β-apiosidase) followed by liberation of the free volatile by the action of a β-glucosidase [14]. Note that β-glucosidase is inactive against disaccharides [27,28]. Although β-glucosidase activity is the crucial final enzyme required for VP release, the *Saccharomyces cerevisiae* enzyme is significantly inhibited by both glucose and ethanol, likely accounting for observations by Caffrey and co-workers that nearly three-quarters of all putative VP-glycosides (as assessed by high-performance liquid chromatography-mass spectrometry; HPLC–MS) survived primary fermentation, with the most substantial changes in concentration occurring during its initial stages [8]. Nevertheless, there is evidence that suggests that the free terpenic profile of wines—typically pleasant aroma compounds which originate from glycosidically-bound precursors comparable to those borne by VPs—depends on the yeast strain used during fermentation [28,29]. These differences could be due not only to strain-specific variation in the activities of the requisite glycosidases [29,30], but also the differential secretion of these enzymes into the grape juice [30], the rate of uptake (into yeast cells) of aroma-glycosides [31], or differences in the expression of pectinolytic enzymes that release cell-wall bound aroma compounds [32]. Using a range of smoke-tainted and control wines analyzed over six years, Ristic and colleagues have demonstrated that VP levels only slightly increased during aging [21]. Interestingly, guaiacyl-glycosides also appeared to increase over the same time period—an observation that complicates the elucidation of the origins of the free VPs. Collectively, these data would suggest that the selection of yeast strains that are poorly able to hydrolyze VP-glycosides, in particular VP-glucosides, during primary fermentation would be a possible way to keep free VP levels low in wines produced from smoke-exposed grapes in which subsequent hydrolysis during aging is expected to be minimal. To lend credence to this potential amelioration strategy, the stability of a range of VP-glycosides towards acid- or enzyme-catalyzed hydrolysis during aging and primary fermentation were evaluated in this study (Figure 1b). In contrast to previous studies [8,10,21], here samples were fortified with known concentrations of 11 different VP-glyconjugates, permitting an assessment of the impact of substituents on the phenyl group (i.e., the aglycone) and of the glycone (i.e., monosaccharide vs. disaccharide). Six different yeast strains, some of which are known to have differential β-glucosidase activities [33], were evaluated with respect to their ability to produce free VPs. Control and smoke-exposed Pinot Noir grapes collected from the same vineyard were used, with the fortification of each set of samples with VP-glycosides permitting a comparison of the release of VPs from a pool naturally stored within grape tissues and also verified glycoconjugates.

## 2. Results and Discussion

### 2.1. Hydrolysis of VP-Glycosides during Aging in a Model Wine

Previous work on the (presumptive) hydrolytic lability of VP-glycosides in wine relied on incurred VP-glycosides [8,21]. While effective, there are confounding variables in this type of experiment that preclude a systematic evaluation of VP-glycoside stability (e.g., microbial vs. chemical hydrolysis, oxidative loses, or colloid association(s)). To overcome these challenges, this study used model wine and commercially-sourced Cabernet Franc wine that were fortified with a suite of VP-glycosides previously synthesized by the authors [34]. The VP-glycosides evaluated included β-d-glucosides of different VPs, as well as several guaiacol-disaccharides (Figure 1b and Table S2) that cover the some of the major forms of VP-glycosides identified in wines made from smoke-tainted grapes [2,5]. The model wine fortifications served as a simplified control system that permitted the exclusion of confounding variables that could exist in real wine including, but not limited to, co-association with other wine components such that the VP-glycosides are not detected using conventional analytical techniques. As well, the absence of any incurred free VPs in the model wine facilitated the absolute quantitation of liberated VPs under a variety of conditions (Table 1). In total, the model wines were treated four different ways after fortification in a preemptive attempt to address potential confounding variables. First, some samples were stored under an argon atmosphere to assess the potential impact of oxidation [35] on the recovery of free VPs. Secondly, to unambiguously rule out microbial metabolism of both free VPs and glycosides thereof, a second set of fortified samples was filtered through a 0.22 μm filter prior to aging. The data indicate that although in many cases traces of free VPs could be recovered from non-aged samples (week 0), likely due to slight traces remaining in the VP-glycoside standards after their synthesis, no significant hydrolysis occurred over 12 weeks of storage at ambient temperature with only two exceptions. First, when added to samples as the monoglucoside, *p*-cresol (*p*-Cre) exhibited a slight but significant (Welch’s test) increase in both non-filtered samples (1.6 and 0.7 ng/g, respectively) over this aging period; this would correspond to less than one percent of the 200 ng/g *p*-Cre-Glc initially added. Secondly, a significant 15.4 ng/g increase in syringol (Syr, supplied as the monoglucoside) was observed in the filtered model wine stored under argon, corresponding to 7.7% of the glycoside being hydrolyzed. Nevertheless, the overall results suggest that at a typical wine pH, VP-glycoside hydrolysis rates are slow, consistent with previous studies.

### 2.2. Hydrolysis of VP-Glycosides during Aging in a Commercial Wine

We next sought to directly evaluate the stability of VP-glycosides in a more complex matrix, a commercially sourced Cabernet Franc wine (Table 2 and Table S2). Among the disaccharide-linked VPs (gentiobiosides of guaiacol (Gua) and Syr, and Gua-primeveroside), intact glycosides were quantitatively recovered after 12 weeks of aging/storage. Previous studies have indicated that VP-disaccharides account for the majority of all VP-glycosides present in smoke-exposed grapes [4,8]. Accordingly, these data suggest that any of these VP-glycosides remaining intact after primary fermentation will remain recalcitrant towards hydrolysis during subsequent aging/storage of wine. Interestingly, some of the monoglucosides exhibited moderate hydrolysis rates. Specifically, both of the 4-alkyl-substituted phenyl-glucosides, i.e., 4-ethylphenyl- and *p*-cresyl-β-d-glucopyranoside—exhibited recoveries ranging from 70 to 84 (average 76) percent in all treatment conditions except those that were both filtered and stored under argon. Minor losses also occurred for the glucosides of Gua, Syr, and 4-methylguaiacol under some treatment conditions. The increased stability of the 6′-substitued glycosides (gentiobiosides/primeverosides are more effectively recovered after aging than their corresponding glucosides even when the VPs moieties are identical) has been previously observed by researchers who have demonstrated that substitution at the 6′ hydroxyl group hinders the formation of the oxacarbenium ion transition state through which the acid-catalyzed hydrolysis of β-glucosides are thought to proceed [36]. Nevertheless, despite some VP-glycosides exhibiting moderate levels of hydrolysis over the period evaluated, the low prevalence of monoglucosides reported in smoke-tainted wines [4,8,34] suggests that the relative increase would not be likely to impact consumer perceptions of smoke taint intensity.

**Figure 2:**
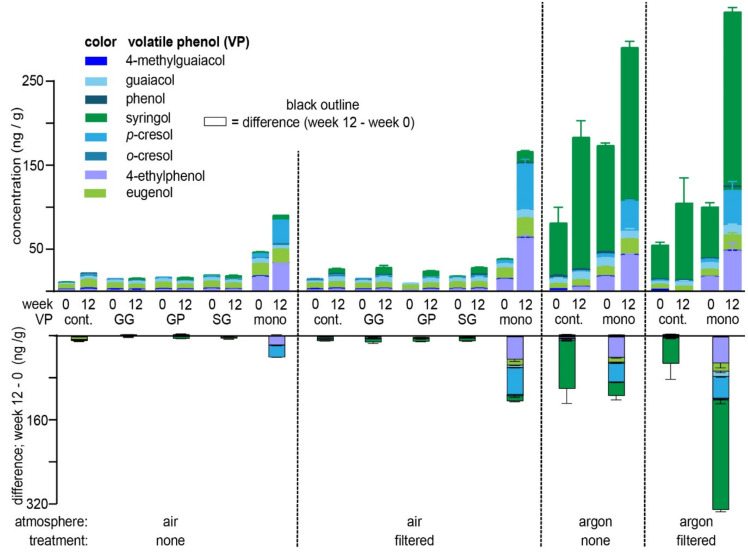
Quantitation of free volatile phenols (VPs) from Cabernet Franc wine that had been fortified with 200 ng/g of VP-glucosides and stored under differing conditions for 12 weeks (top panel). Note that GG, GP, SG, and mono refer to guaiacol-gentiobioside, guaiacol-primeveroside, syringol-gentiobioside and VP-monoglucosides, respectively; cont. = control, i.e., unfortified samples. Error bars depict the standard error of the mean. Bars with black outlines (the lower panel) were calculated by determining the difference between free VP concentrations quantitated at week 12 and week 0; error bars for the difference in VP concentrations denote the sum of the errors in quadrature. Note the top and bottom panels have different *y*-axis scales.

Attempts to achieve a mass balance equating a loss in VP-glycosides (Table 2) with a gain in free VPs during aging (Figure 2) revealed that VPs were released into the wine from endogenous pools that were, in some instances, unrelated to the model VP-glycosides with which samples were fortified (see Figure 1 and Table S1). Firstly, in all four treatment conditions, the wines fortified with the monoglucosides of 4-ethylphenol (4-EP) and *p*-cresol (*p*-Cre) exhibited significant increases in the free VP after 12 weeks of storage. With the exception of the samples stored under air without prior filtration, the increase in free 4-EP and *p*-Cre (i.e., the difference between VPs measured at week 12 and 0) exceeded the fraction of the corresponding loss in VP-glucosides (Table 2); this discrepancy is especially notable for the samples that had been filtered and stored under argon where both 4-EP- and *p*-Cre-glucosides were quantitatively recovered, although 50 and 42 ng/g increases in the free VPs were, respectively observed. Even more striking is the notable increase in free syringol (Syr) in the argon-treated samples. For example, control wines which were not fortified with any VP-glycosides exhibited 93 and 51 ng/g increases in Syr in the non-filtered and filtered wines, respectively; that these changes were not detectable in samples under an air atmosphere is possibly due to the oxidative loss of free Syr within these samples. In VP-glycoside-fortified wines stored under argon, the filtered sample exhibited an astonishing 152 ng/g increase in free Syr after a 12 week period over which the Syr-monoglucoside was stable, as indicated by its nearly quantitative recovery. Thus, given that the VP-β-d-glucosides and disaccharides thereof exhibited limited hydrolysis in both model and actual wine, the origin of the free 4-EP, *p*-Cre and Syr in these wine samples suggests the existence of at least some “bound” VP-precursors, that are not simple glycosides in commercial, untainted wines.

### 2.3. Hydrolysis of VP-Glucosides during Primary Fermentation with Different Yeast Strains

**Figure 3:**
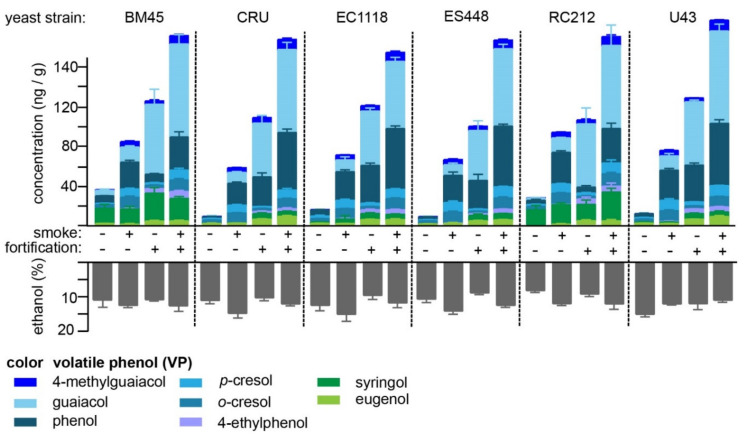
Concentrations of volatile phenols (VPs) in wines produced by different yeast strains from smoke-exposed or control Pinot Noir grapes with and without fortification with VP-monoglucosides. All VP-monoglucosides were added to grape samples at a concentration of 200 ng/g. The lower, gray histograms depict the concentration of ethanol measured at the end of primary fermentation.

As noted earlier, Caffrey and co-workers [8] have previously demonstrated that VP-glycosides (although only putatively identified, presumably these are glucosides) survive primary fermentation, with approximately three-quarters of those present in the juice recovered in the resulting wine. These data are consistent with results reported by Ristic and co-workers who compared the abilities of eight wine yeast strains to hydrolyze guiaical glycoconjugates in smoke-exposed Grenache grape musts [22]. However, the increase in some VPs detectable simply upon storage of non-smoke-tainted wines (Figure 2) suggests in-grape sources of these compounds that are not typical β-d-glucosides nor necessarily linked to grape smoke exposure. Furthermore, previous research by Szeto and co-workers [10] has demonstrated that VPs may be sequestered into Cabernet Sauvignon grape tissues as strong acid-labile conjugates that are not VP-glucosides, while Noestheden and co-workers have likewise demonstrated that smoke-exposed Pinot Noir grapes trap VPs as acid- [34] or base-labile [11] conjugates that are not known VP-glucosides. Thus, an experiment was designed to compare the capacity of different yeast strains to hydrolyze known VP-glucosides and as-yet uncharacterized in-grape VP storage forms. More specifically, Pinot Noir grapes that had been exposed to smoke during a previous field study, and matched controls from the same year/vineyard, were inoculated with six different strains of yeast with or without fortification with a range of VP-monoglucosides (Figure 3 and Figure S2, Table 3). Because VP concentrations may exhibit an additive effect with respect to their perception in wines [9], VPs recovered after fermentation are displayed both in aggregate (Figure 3) and separately (Table 3 and Figure S2).

No discernable trends were apparent to suggest that either VP-glucoside fortification or the use of smoke-exposed must influenced the fermentation activity of the yeast strains evaluated. Each yeast strain tested released minor concentrations of VPs into the wine, with aggregate totals varying between 11 (ES448) and 38 ng/g (BM45; Figure 3) when using must prepared from non-smoke-exposed grapes for the fermentation. Similarly, when smoke-exposed grape musts were fermented, unequal increases in VP concentrations were observed, ranging from 59 (Cru) to 97 ng/g (RC212)—a 1.6-fold difference between the highest and lowest yeast strains. The largest contributors to the increase in VPs observed, in comparing smoke-exposed and control grapes, were noted for phenol and guaiacol (Table 3 and Figure S2). When the control, non-smoked grapes fortified with 200 ng/g of VP-monoglucosides were fermented, a higher concentration of VPs was detected than using the solely smoke-exposed grapes for all yeast strains, albeit the relative difference between the highest and lowest concentrations (total of all VPs)—105 (ES488) to 131 ng/g (BM45), 1.2-fold—was not as great as that observed for the smoked grapes. For the VP-monoglucoside fortified samples, guaiacol and phenol exhibited the largest increase in concentration after fermentation, exhibiting 23- and 5-fold increases in the VP-glucoside-fortified samples relative to the controls, respectively (Table 3). In all cases of fortification of VP-glucosides into control grape must, the extent of VP hydrolysis substantially exceeded that observed for the spontaneous acid-catalyzed hydrolysis in model wine fortified at an equivalent concentration (Figure 2), clearly implicating the action of yeast glucosidases in the increased concentration of these compounds. For example, the maximum change in guaiacol concentration during 12 weeks of storage (observed for the filtered samples stored under air; Table S2) was 5.8 ng/g whereas when must was fortified with guaiacol-β-d-glucoside, an average (across all six strains tested) increase of 60 ± 3.4 ng/g guaiacol was observed in the resulting wines. Finally, an additive effect of using both smoke-exposed and VP-glucoside-fortified grape musts was most notable for phenol and 4-methylguaiacol, although completely absent for both *o*- and *p*-cresol (Figure S2). These data indicate that β-d-glucosides of phenol and guaiacol are most efficiently cleaved by yeast glucosidases during fermentation, and that if other analogues of these VPs do exist within smoke-exposed grapes (whether as disaccharides or as yet uncharacterized conjugates) these additional VPs are not inhibitory.

The data do suggest that at least some of the VPs linked to smoke taint are stored within grapes as conjugates that are not simple glycosides. For example, two yeast strains, BM45 and RC212, yielded high (15–29 ng/g) and essentially invariant concentrations of syringol, regardless of both smoke exposure and fortification. As β-glucosidase action is currently thought to be the last, necessary step for free VP release (Figure 1a), the consistent concentrations of syringol noted for BM45 and RC212 must have originated from a source that was not a β-glucoside or disaccharide possessing a reducing end β-glucoside moiety. Note that it is unlikely that this free syringol could have been in the juice prior to fermentation as wines produced by two yeast strains (Cru and ES44) from unfortified, non-smoked grapes contained syringol levels that were below the method detection limit for this VP (Table 3 and Figure S2). Likewise, both *o*- and *p*-cresol, were not elevated above unfortified control fermentations for any yeast strain evaluated when these VPs were added to musts at 200 ng/g of their corresponding glucosides (Figure S2). However, conducting the fermentations with the smoked grape samples led to a minimum 2-fold increase in both cresols in all instances, again suggesting that these cresols may not be present in the smoke-exposed grapes as canonical β-glucosides. Significant yeast strain-specific differences were also noted for phenol. For example, BM45 yielded the greatest concentration of phenol (8.3 ng/g) from control grapes, a value that remained essentially constant (9.6 ng/g) in grapes fortified with phenol-β-d-glucoside (*i.e.,* there is no evidence for BM45-derived β-glucosidase activity against this glycoside), albeit all other strains except RC212 effectively produced three- to four-fold more free phenol when fortified prior to fermentation. However, both RC212 and BM45 did produce comparable (to the other strains) concentrations of phenol from smoke-exposed grapes, suggesting that phenol may not be present in both control and smoke-exposed grapes solely as a β-d-glucoside. Note that the aglycone (i.e., VP) does influence enzyme-catalyzed glucoside hydrolysis as, in contrast to phenol, both RC212 and BM45 yielded substantial increases in guaiacol and syringol concentrations in fermentations fortified with the respective glucosides of these VPs. In summary, these data demonstrate that commercial wine yeasts may differ with respect to their ability to hydrolyze β-glucosides of smoke taint-associated VPs during fermentation and that these differences, especially in the case of RC212 and BM45, are due in part to the nature of the aglycone. These data also provide more indirect evidence that at least some smoke taint-associated VPs (i.e., syringol) are normally present in grapes and that not all VPs derived from smoke are necessarily present in grapes as β-glucose-linked glycosides.

In conclusion, with smoke taint remaining an issue of concern for the global wine industry, methods to limit the hydrolysis of VP-glycosides during the fermentation and aging of wine offer one method of preventing the release of free VPs positively associated with the intensity of smoke taint in the finished wines. Herein, by tracking the production of free VPs in a model wine fortified with a range of VP-glycosides, we have demonstrated that at the typical acidic pH values of normal red wines, minimal hydrolysis occurs over a 12 week period with the single exception of syringol-β-d-glucopyranoside, which is unlikely to be a strong contributor to smoke taint given its high aroma threshold. Meanwhile, a similar experiment conducted in an actual red wine matrix demonstrated moderate losses in two VP-glucosides (4-ethylphenol and *p*-cresol) with a concomitant increase in the concentrations of the free aglycones. Nevertheless, VP-disaccharides, which have been previously identified to be the major in-grape storage forms of VPs, were found to be unchanged in both model and actual wine over this time period. Given the apparent stability of VP-disaccharides, an experiment was designed to evaluate the differences of several wine-making yeast strains with respect to their ability to enzymatically catalyze the final step in the liberation of free VPs into wines, namely hydrolysis of the β-glucosidic bond to the VP. Consistent with our simulated aging experiment using Cabernet Franc wine (in which free syringol was detectable at equivalent levels in both unfortified and fortified samples stored under argon), our results indicated that syringol may also be present in Pinot Noir grapes as a bound VP that does not contain a β-linked glucose residue (as two of six yeast strains produced moderate amounts of free syringol, independent of both smoke exposure and fortification). Both smoke exposure and fortification with VP-monoglucosides led to increases in the free VPs quantitated in wines made from all yeast strains (an effect that was additive for most VPs except both of the cresols and syringol), with the majority of this increase attributable to guaiacol followed by phenol. Yeast strain-specific differences in VP production were most notable for the phenol and two (RC212 and BM45) were unable to hydrolyze the β-d-glucoside of phenol although they were comparable to other strains when smoked-exposed grapes were used for fermentation. Nevertheless, further research into how VPs are biochemically sequestered within grape tissues and the differential capacity of *S. cerevisiae* strains to hydrolyze these aroma precursors, especially those that are not simply β-glucosides (which are likely a minority) is required before yeast selection can be considered a verified strategy for smoke taint mitigation in wine.

## 3. Materials and Methods

### 3.1. Chemicals and General Details

The following chemicals were purchased from Sigma Aldrich (Saint Louis, MO, USA): HPLC-grade methanol (MeOH), isopropanol (IPA), acetonitrile (ACN), hexane, ethyl acetate (EtOAc), hydrochloric acid (HCl), acetic acid, sodium chloride, ammonium formate, potassium phosphate, magnesium sulfate, glucose, fructose, tartaric acid, malic acid, 2-methoxyphenol (guaiacol), 2,6-dimethoxyphenol (syringol), 2-methoxy-4-methylphenol (4-methylguaiacol), 2-methoxy-4-ethylphenol (4-ethylguaiacol), 4-ethylphenol, 2-methoxy-4-allylguaiaicol (eugenol), 2-methylphenol (*o*-cresol), 4-methylphenol (*p*-cresol), *d_3_*-guaiacol, *d_4_*-4-ethylphenol, *d_5_*-4-ethylguaiacol. *d_7_*-*o*-Cresol and *d_7_*-*p*-cresol were purchased from Toronto Research Chemicals (Toronto, ON, Canada). All chemicals were used as received. The syntheses of *d_3_*-syringol [11] and model VP-glycosides (*d_3_*-guaiacyl-β-d-glucopyranoside, guaiacyl-β-d-glucopyranoside, syringyl-β-d-glucopyranoside, eugenyl-β-d-glucopyranoside, 4-methylguaiacyl-β-d-glucopyranoside, 4-ethylphenyl-β-d-glucopyranoside, *p*-cresyl-β-d-glucopyranoside, guaiacyl-primeveroside, guaiacyl-gentiobioside, syringyl-gentiobioside, and guaiacyl-rutinoside) are reported elsewhere [11,34].

Type 1 water (18 MΩ·cm) was provided by a Barnstead E-Pure water purification system (Thermo Fisher Scientific; Waltham, MA, USA). A Mettler Toledo FE20 FiveEasy pH meter was used to measure pH. An Allegra X-12R centrifuge (Beckman Coulter, Mississauga, ON, Canada) and a Savant SPD121P SpeedVac concentrator connected to a Savant RVT5105 refrigerated vapor trap (Thermo Fisher Scientific, Waltham, MA, USA) were used for sample preparation. Strata-X SPE (1 mL/30 mg) cartridges were purchased from Phenomenex (Torrance, CA, USA). Four mL borosilicate glass vials with phenolic caps and polyvinyl-faced pulp liners were used as received (VWR, Mississauga, ON, Canada). All solvents used were HPLC-grade.

### 3.2. Stock and Calibration Solutions

Concentrated VP and ISTD stock solutions were prepared in IPA (*d_3_*-syringol in MeOH) at 1.0–10.0 mg/mL. An ISTD intermediate stock solution (*d_3_*-guaiacol, *d_4_*-4-ethylphenol, *d_5_*-4-ethylguaiacol, *d_7_*-*o*-cresol, *d_7_*-*p*-cresol and *d_3_*-syringol) was prepared at 5 μg/mL in IPA. This solution was added to all calibration samples and extracts to a working concentration of 50 ng/mL. Nine intermediate calibration stock solutions containing all the target VPs (guaiacol, 4-methylguaiacol, 4-ethylguaiacol, 4-ethylphenol, *o*-cresol, *p*-cresol, syringol and eugenol) were prepared in IPA from 0.1–100 μg/mL. Calibration samples from 1–1000 ng/mL were prepared fresh daily by diluting the intermediate calibration stock solutions 100-fold into 1:1 hexane:EtOAc containing ISTD (50 ng/mL). Individual model glycoside stock solutions were prepared in IPA at 1 mg/mL. An intermediate mixture of all the β-d-glucopyranosides was prepared in IPA at 100 μg/mL. Concentrated and intermediate stock solutions were stored at −20 °C for up to 12 months.

### 3.3. Spontaneous Hydrolysis

All samples were prepared under sterile conditions. A commercially purchased Cabernet Franc (2015 Okanagan, BC, Canada) and a model wine matrix were prepared by sterile filtering (0.22 µm) 100 mL of each matrix and aliquoting another 100 mL of each unaltered. The model wine solution was composed of the following: ethanol (12% *v*/*v*), tartaric acid (5.0 g/L), malic acid (3.5 g/L), acetic acid (0.6 g/L), glucose (2.0 g/L), fructose (2.0 g/L), NaCl (0.2 g/L), KH_2_PO_4_ (2.0 g/L) and MgSO_4_ (0.4 g/L). For each of these conditions, the matrices were then either carried forward without additional processing, or they were fortified with model VP-glycosides to 200 ng/mL (to enable correlation of free VPs with loss of specific VP-glycosides three sets of samples fortified with VP-glycosides were prepared to accommodate those that had the same VP attached to different sugars [e.g., guaiacyl-primeveroside and guaiacyl-gentiobioside]). These four treatment groups were split again (for a total of eight treatment groups) by storing samples under air or under argon. A schematic of this experimental design can be found in Figure S1. Samples from all treatment conditions were aliquoted (2 mL) directly into 4 mL borosilicate glass vials that had been sterilized by exposure to 70% (*v*/*v*) EtOH for 15 min. All treatment groups were prepared in triplicate and stored under ambient conditions protected from light. After aliquoting, samples stored under argon had the headspace purged with argon (99.999%) for 15 s prior to tightening the closures. Samples were analyzed at the start of the experiment (*t* = 0 d) and after three months (*t* = 90 d). All samples were analyzed for free VPs and the commercial wine samples were analyzed for intact VP-glycosides (*vide infra*).

### 3.4. Vinification

Control and smoke-exposed Pinot Noir berries were acquired from 2017 field trials conducted in the Okanagan Valley of British Columbia, Canada near 49.7694° N, 119.5306° W. In brief, vines were exposed to smoke produced from 1.5 kg of *Pinus ponderosa* fuel (soil organic matter, bark and needles) twice over a period of two days, each smoke exposure lasting for one hour. The polyethylene enclosure used to contain the smoke accommodated 12 vines. Control grapes were harvested from adjacent vines. Smoke exposure occurred approximately seven days post-*verasison*. Further details of the field trial experimental design were previously published by Noestheden et al. [13]. Six commercial strains of yeast were utilized to ferment the control and smoke-exposed Pinot Noir berries, with each treatment group further split into one set of unfortified ferments (*n* = 3) and a set of ferments fortified with model VP-glycosides (200 ng/mL; *n* = 3). The list of VP-glycosides used to fortify the ferments is included in Table S1. The strains of yeast included Lallemand BM45, Fermol Premier Cru, Lallemand EC1118, Vitilevure 3001, Uvaferm 43 and Enartis Ferm ES 488. Strain-typing [37] was performed on all cultures to confirm the use of genetically distinct strains.

Yeast cultures were prepared by streak-plating onto agar and incubating at 28 °C. After 48 h, a subsample of the yeast was transferred to 45 mL of yeast extract peptone dextrose media (10% yeast extract, 10% bacterial peptone, 20% dextrose; YEPD), followed by incubation at 28 °C. After 24 h, the optical density of the inoculum was adjusted to 2.0 absorbance units (600 nm) with sterile water.

Micro-scale wines (*n* = 3/treatment group) were prepared as follows: Erlenmeyer flasks (500 mL) were sanitized by exposure to 20 mL of 5% sodium metabisulfite solution for one hour and rinsed thoroughly with water. For each of the berry samples from control and smoke-exposed vines, 100 g aliquots of frozen berries were weighed into a sanitized Erlenmeyer flask and 5 mL of a 1% sodium metabisulfite solution was added. The berries were thawed for 24 h at 4 °C, crushed with a spatula and, where necessary, the pH of the juice was adjusted to 3.6–3.8 by the addition of 10% aqueous tartaric acid (<500 μL). Prior to adding the inoculum, one set of samples was fortified with the model VP-glycosides (200 ng/g). The resulting musts were inoculated with yeast culture (5 mL). Flasks were then fitted with air locks and fermentations proceeded with twice daily agitation. The progress of fermentation was monitored by collecting daily measurements of the total soluble solids (°Bx; Table S3). Fermentation was halted by removing yeast cells via centrifugation (3000× *g* for 10 min). The final wines obtained after centrifugation were stored in glass at 2–8 °C under argon prior to analysis. Each ferment yielded 30–50 mL of wine.

The ethanol content of each wine was assessed using an Ethanol Assay Kit (Megazyme, Irishtown, County Wicklow, Ireland), with external calibration from 20–150 µg/mL used to quantitate the absolute ethanol concentrations. The plate reader was a Varioskan Lux (Thermo Fisher Scientific, Waltham, MA, USA), with absorbance readings taken at 340 nm.

### 3.5. Sample Preparation and Analysis

For GC–MS/MS analyses, free VPs were extracted from the wines and analyzed exactly as described by Noestheden et al. [13]. Briefly, wines or model wines were fortified with deuterated ISTDs before they were saturated with NaCl and extracted into a 1:1 (*v*:*v*) mixture of hexanes and ethylacetate. After centrifugation (3000× *g*, 2 min), 1 mL of the organic layer was transferred into a GC vial for analysis using a Trace 130 gas chromatograph equipped with a TSQ9000 triple quadrupole mass spectrometer (both from Thermo Fisher Scientific, Waltham, MA, USA). A Zebron ZB-5MSplus (30 m × 0.25 mm × 0.25 μm column (Phenomenex; Torrence, CA, USA) was used for VP separation using helium (1.5 mL) as the carrier gas. GC–MS/MS system parameters were exactly the same as previously reported [13]. The extraction and analysis of the intact VP-glycosides using uHPLC-QToF was performed as described previously by Noestheden et al. [34]. In brief, all wine samples were dried in vacuo and re-dissolved in an equivalent volume of water before fortification with at d_3_-guaiacol-β-d-glucopyranoside ISTD prior to extraction using a Strata-X (Phenomenex) solid phase extraction cartridge. The organic eluate (40% ACN) was dried and resuspended in 1 mL H_2_O before analysis using an Agilent (Santa Clara; CA, USA) 1290 Infinity HPLC system and an Agilent 6530 QToF with a Jet Stream electrospray ionization source operating in negative ion mode. A 20 μL sample was injected onto a Luna Omega C18 (40 × 2.1 mm, 1.6 μm) column. A list of VP-glycosides, formulae, and HPLC retention times is found in Table S1; more detailed method parameters may be found elsewhere [34].

### 3.6. Data Acquisition and Processing

GC–MS/MS data were processed using the Xcalibur (v 3.0.63) and TraceFinder (v 3.2.512.0) software packages (Thermo Scientific, Waltham, MA, USA). The uHPLC-QToF data acquisition and processing were carried out using the MassHunter Workstation software suite (Agilent Technologies, Santa Clara, CA, USA), with version numbers as follows: Data Acquisition Workstation (v B.06.01, Service Pack 1) Qualitative Analysis (v B.07.00, Service Pack 2) and Quantitative Analysis (v B.07.00). Data reduction was performed using Microsoft Excel (Microsoft Corporation, Redmond, WA, USA). Levene’s test for homogeneity, as well as Kruskal–Wallis and Tukey’s HSD *post hoc* tests were performed on GC–MS/MS data within treatment groups. A two-tailed Welch’s test of unequal variance was also done to compare differences in free VP recovery in both model wine and Cabernet Franc wine after 12 weeks of spontaneous hydrolysis versus immediately after VP fortification. All statistical tests were performed in RStudio (RStudio, Inc., version 3.5.1, Boston, MA, USA). All tests were considered significant at *p* ≤ 0.05. For VP values that were below the limit of detection, non-detects were replaced by the lowest detected value of a particular compound divided by five. This allowed cases where there were below triplicate values for a specific compound to be excluded from statistical analyses. The detailed procedure and explanation of this method is explained elsewhere [38].

## Figures and Tables

**Table 1:** Recovery of free VPs from model wine matrix (pH 3.6) fortified with VP-glycosides (200 ng/g) after 12 weeks of spontaneous hydrolysis.

VP-Glycoside	Atmosphere/Treatment ^1,2,3^							
	Air/Not Filtered		Air/Filtered		Argon/Not Filtered		Argon/Filtered	
	Week 0	Week 12	Week 0	Week 12	Week 0	Week 12	Week 0	Week 12
4-EP-Glc	15.2 ± 0.70	17.3 ± 0.65	14.4 ± 0.77	16.6 ± 0.69	11.3 ± 0.23	13.5 ± 1.10	9.0 ± 3.39	14.6 ± 1.27
4-MG-Glc	-	-	-	-	-	-	-	-
Eug-Glc	6.2 ± 0.61	9.2 ± 0.95	7.1 ± 0.60	7.6 ± 0.69	4.3 ± 0.24	4.8 ± 0.24	4.9 ± 3.06	8.0 ± 0.70
Gua-Glc	1.0	1.4 ± 0.19	-	1.2	1.1	1.6 ± 0.14	-	1.3 ± 0.18
*o*-Cre-Glc	-	-	1.2	1.3 ± 0.39	-	-	-	-
*p*-Cre-Glc	**2.8 ± 0.27**	**4.4 ± 0.32**	2.7 ± 0.17	3.1 ± 0.17	**2.4 ± 0.06**	**3.1 ± 0.13**	2.5 ± 1.08	3.3 ± 0.23
Phe-Glc	-	-	-	-	-	-	-	-
Syr-Glc	20.4 ± 0.92	23.9 ± 1.67	18.6 ± 0.72	28.5 ± 3.65	22.3 ± 0.47	24.1 ± 0.97	**13.9 ± 3.01**	**29.3 ± 3.16**
Gua-Gent	-	-	-	-	n.a.		n.a.	
Gua-Prim	-	-	-	-	n.a.		n.a.	
Syr-Gent	2.1 ± 0.44	3.1 ± 0.35	2.3 ± 0.13	3.3 ± 0.56	n.a.		n.a.	

Notes: ^1^ Fortified model wines (see Table S1) were stored under an atmosphere of air or argon gas; when indicated, samples were filtered through a 0.22 μm filter prior to storage. ^2^ Data are provided as the mean VP concentrations (in ng/g) recovered from each sample ± the standard error of the mean (SEM). A dash indicates that the concentration of the indicated VP was below the limit of quantitation for all replicates. ^3^ Values in bold represent a statistically significant difference (two-tailed Welch’s test of unequal variance, *n* = 3/condition, α = 0.05) in comparison to the same samples analyzed immediately after fortification of the model wine. The following abbreviations are used: Glc = β-d-glucopyranoside, Gent = gentiobioside, and Prim = primeveroside; 4-EP = 4-ethylphenol, 4-MG = 4-methylguaiacol, Eug = eugenol, Gua = guaiacol, *o*-Cre = *o*-cresol, *p*-Cre = *p*-cresol, Phe = phenol, and Syr = syringol; n.a. = not applicable.

**Table 2:** Recoveries (%) of intact VP-glycosides from fortified (200 ng/g) Cabernet Franc wines after 12 weeks of spontaneous hydrolysis.

VP-Glycoside	Atmosphere/Treatment ^1,2^			
	Air/Not Filtered	Air/Filtered	Argon/Not Filtered	Argon/Filtered
4-EP-Glc	70.3 ± 8.24	76.0 ± 3.64	77.1 ± 2.83	99.8 ± 4.88
4-MG-Glc	84.8 ± 3.31	120.7 ± 7.65	121.3 ± 9.82	89.4 ± 12.45
Eug-Glc	84.8 ± 4.28	99.5 ± 8.89	109.4 ± 5.22	103.5 ± 8.46
Gua-Glc	93.9 ± 5.98	82.3 ± 4.89	112.0 ± 5.58	92.7 ± 7.72
*p*-Cre-Glc	72.5 ± 7.57	84.0 ± 4.23	75.0 ± 4.51	105.5 ± 5.54
Syr-Glc	90.9 ± 5.40	88.3 ± 4.13	80.3 ± 5.30	95.8 ± 5.74
Gua-Gent	102.2 ± 7.24	104.9 ± 7.07	n.a.	n.a.
Gua-Prim	106.9 ± 17.82	102.3 ± 6.21	n.a.	n.a.
Syr-Gent	102.4 ± 6.05	118.4 ± 3.72	n.a.	n.a.

Notes: ^1^ Fortified wines were stored under an atmosphere of air or argon gas; when indicated, samples were filtered through a 0.22 μm filter prior to storage. ^2^ Mean VP-glycoside concentrations were measured in triplicate after 0 and 12 weeks of spontaneous hydrolysis; percent recoveries (100% × week 12/week 0) are reported ± the standard error of the mean (SEM). The following abbreviations are used: Glc = β-d-glucopyranoside, Gent = gentiobioside, and Prim = primeveroside; 4-EP = 4-ethylphenol, 4-MG = 4-methylguaiacol, Eug = eugenol, Gua = guaiacol, *p*-Cre = *p*-cresol, and Syr = syringol. n.a. = not applicable.

**Table 3:** Free VPs quantitated in Pinot Noir wines fermented with six commercial strains of *S. cerevisiae*. Treatment groups included grape musts that were unfortified/fortified with VP-monoglucosides (200 ng/g), as well as musts that were unsmoked/smoked.

Strain	Fortified	Grapes	Concentration (ng/g) ^1,2^							
			4-MG	Gua	Phe	*p*-Cre	*o*-Cre	4-EP	Syr	Eug
EC1118	N	ctrl	-	1.3 ± 0.02 ^c^	6.45 ± 0.49 ^ab^	3.0 ± 0.44 ^a^	3.5 ± 0.07 ^ab^	-	1.3 ± 0.03 ^b^	1.4 ± 0.04 ^ab^
RC212	N	ctrl	-	4.4 ± 0.60 ^b^	5.1 ± 0.66 ^bc^	2.1 ± 0.14 ^ab^	3.3 ± 0.05 ^abc^	-	15.3 ± 2.36 ^a^	-
BM45	N	ctrl	-	6.9 ± 0.54 ^a^	8.3 ± 0.16 ^a^	1.8 ± 0.01 ^b^	3.6 ± 0.17 ^a^	-	15.8 ± 2.20 ^a^	1.2 ± 0.03 ^ab^
U43	N	ctrl	-	1.4 ± 0.02 ^c^	4.4 ± 0.48 ^cd^	1.7 ± 0.14 ^b^	3.4 ± 0.10 ^abc^	-	1.2 ± 0.10 ^b^	1.6 ± 0.04 ^a^
ES488	N	ctrl	-	1.1 ± 0.04 ^c^	3.8 ± 0.03 ^cd^	1.5 ± 0.12 ^b^	3.0 ± 0.03 ^c^	-	-	1.1 ± 0.06 ^ab^
Cru	N	ctrl	-	1.2 ± 0.48 ^c^	2.8 ± 0.27 ^d^	1.5 ± 0.08 ^b^	3.1 ± 0.09 ^bc^	-	-	0.9 ± 0.33 ^bc^
EC1118	Y	ctrl	5.1 ± 0.32 ^a^	58.1 ± 1.50 ^a^	39.4 ± 1.41 ^a^	3.5 ± 0.33 ^ab^	3.3 ± 0.09 ^a^	4.2 ± 0.34 ^ab^	7.2 ± 0.28 ^c^	5.2 ± 0.25 ^abc^
RC212	Y	ctrl	3.6 ± 0.50 ^a^	67.4 ± 9.71 ^a^	6.9 ± 0.93 ^c^	3.1 ± 0.31 ^abc^	3.3 ± 0.23 ^a^	6.2 ± 1.09 ^a^	16.7 ± 2.42 ^b^	3.6 ± 0.56 ^cd^
BM45	Y	ctrl	3.4 ± 0.59 ^a^	74.5 ± 9.2 ^a^	9.6 ± 0.48 ^c^	3.0 ± 0.16 ^abc^	3.4 ± 0.24 ^a^	5.4 ± 0.67 ^a^	28.6 ± 3.13 ^a^	3.5 ± 0.50 ^d^
U43	Y	ctrl	4.4 ± 0.07 ^a^	67.5 ± 0.87 ^a^	37.5 ± 1.30 ^ab^	3.9 ± 0.05 ^a^	3.2 ± 0.05 ^a^	5.1 ± 0.09 ^a^	7.5 ± 0.15 ^c^	5.9 ± 0.04 ^a^
ES488	Y	ctrl	3.7 ± 0.11 ^a^	53.8 ± 5.67 ^a^	29.9 ± 3.59 ^b^	2.5 ± 0.13 ^bc^	3.0 ± 0.12 ^a^	2.2 ± 0.19 ^b^	5.7 ± 0.64 ^c^	4.3 ± 0.23 ^bcd^
Cru	Y	ctrl	5.2 ± 0.49 ^a^	57.4 ± 4.43 ^a^	30.3 ± 2.34 ^ab^	2.5 ± 0.02 ^c^	3.1 ± 0.14 ^a^	2.4 ± 0.32 ^b^	5.8 ± 0.35 ^c^	5.8 ± 0.16 ^ab^
EC1118	N	smoked	4.7 ± 0.13 ^a^	13.6 ± 1.60 ^ab^	29.67 ± 1.22 ^a^	8.4 ± 0.62 ^ab^	10.0 ± 0.21 ^a^	0.5 ± 0.27 ^a^	4.8 ± 1.90 ^b^	1.2 ± 0.04 ^a^
RC212	N	smoked	5.4 ± 0.23 ^a^	16.3 ± 0.5 ^ab^	32.9 ± 1.01 ^a^	9.8 ± 0.29 ^ab^	11.3 ± 0.35 ^a^	0.5 ± 0.33 ^a^	19.7 ± 0.85 ^a^	0.8 ± 0.30 ^a^
BM45	N	smoked	5.3 ± 0.39 ^a^	17.0 ± 0.54 ^a^	28.3 ± 1.20 ^ab^	8.7 ± 0.46 ^ab^	11.5 ± 0.67 ^a^	0.5 ± 0.30 ^a^	15.7 ± 1.30 ^a^	0.5 ± 0.33 ^a^
U43	N	smoked	5.9 ± 0.42 ^a^	16.3 ± 1.23 ^ab^	31.1 ± 1.13 ^a^	10.9 ± 0.82 ^a^	11.2 ± 0.50 ^a^	0.8 ± 0.29 ^a^	1.8 ± 0.05 ^b^	1.6 ± 0.09 ^a^
ES488	N	smoked	5.2 ± 0.39 ^a^	12.5 ± 0.77 ^b^	27.9 ± 2.02 ^ab^	10.0 ± 0.83 ^ab^	10.8 ± 0.67 ^a^	0.5 ± 0.30 ^a^	-	1.0 ± 0.41 ^a^
Cru	N	smoked	4.8 ± 0.10 ^a^	12.6 ± 0.18 ^ab^	23.1 ± 0.84 ^b^	7.9 ± 0.34 ^b^	9.5 ± 0.22 ^a^	-	-	1.3 ± 0.05 ^a^
EC1118	Y	smoked	9.34 ± 0.72 ^a^	71.6 ± 2.15 ^a^	62.3 ± 1.60 ^a^	9.7 ± 0.37 ^a^	11.2 ± 0.47 ^a^	5.0 ± 0.29 ^ab^	6.4 ± 0.09 ^c^	5.3 ± 0.41 ^b^
RC212	Y	smoked	9.1 ± 0.92 ^a^	88.2 ±12.49 ^a^	35.5 ± 3.23 ^b^	11.5 ± 1.73 ^a^	12.6 ± 0.82 ^a^	6.7 ± 1.92 ^ab^	29.3 ± 2.12 ^a^	4.2 ± 0.81 ^b^
BM45	Y	smoked	8.1 ± 0.77 ^a^	98.7 ± 5.71 ^a^	34.7 ± 3.10 ^b^	10.9 ± 0.98 ^a^	11.6 ± 0.83 ^a^	7.9 ± 0.59 ^a^	22.9 ± 1.01 ^b^	4.2 ± 0.41 ^b^
U43	Y	smoked	10.7 ± 0.26 ^a^	99.4 ± 3.97 ^a^	63.6 ± 2.19 ^a^	11.5 ± 0.07 ^a^	10.3 ± 0.26 ^a^	5.9 ± 0.27 ^ab^	9.0 ± 0.54 ^c^	8.7 ± 0.58 ^a^
ES488	Y	smoked	8.9 ± 0.47 ^a^	82.8 ± 2.34 ^a^	62.8 ± 1.20 ^a^	12.2 ± 0.26 ^a^	11.7 ± 0.57 ^a^	4.8 ± 0.17 ^ab^	7.2 ± 0.10 ^c^	5.5 ± 0.29 ^b^
Cru	Y	smoked	10.1 ± 0.71 ^a^	88.1 ± 4.07 ^a^	58.9 ± 2.11 ^a^	8.8 ± 0.91 ^a^	10.2 ± 0.30 ^a^	3.5 ± 0.46 ^b^	7.5 ± 0.33 ^c^	8.1 ± 0.56 ^a^

Notes: ^1^ Volatile phenol concentrations are reported ± the standard error of the mean (SEM) of triplicate fermentations. ^2^ Statistical differences observed between yeast strains within a single treatment group (Kruskal–Wallis and *post hoc* Tukey’s HSD tests, α = 0.05) are indicated by the different letter codes. Dashes indicate concentrations that were below the limit of quantitation. The following abbreviations are used: 4-MG = 4-methylguaiacol; Gua = guaiacol; Phe = phenol; *p*-Cre = *p*-cresol; *o*-Cre = *o*-cresol; 4-EP = 4-ethylphenol; 4-EG = 4-ethylguaiacol; Syr = syringol; Eug = eugenol.

## Supplementary Materials

The following are available online, Figure S1: Spontaneous hydrolysis experimental design; Table S1: Model VP-glycosides used to fortify ferments; Table S2: Recovery of free VPs from commercial Cabernet Franc wine (pH 3.4) fortified with VP-glycosides (200 ng/g) after 12 weeks of spontaneous hydrolysis; Table S3: Change in total soluble solids (°Bx) as a metric of fermentation progress (days); Figure S2: Comparison of the concentration (ng/g) of VPs released by different yeast strains after primary fermentation.

## Sample Availability

Samples of the compounds are not available from the authors.
