# Supplementary material for: Glycosidically-Bound Volatile Phenols Linked to Smoke Taint: Stability during Fermentation with Different Yeasts and in Finished Wine
Source: Molecules. 2021 Jul 27;26(15):4519. doi: 10.3390/molecules26154519 (PMC8347507; doi:10.3390/molecules26154519)
Supplement: Supplementary file 1 [file molecules-26-04519-s001.zip › molecules-1292893-supplementary.pdf]

# Glycosidically-Bound Volatile Phenols Linked to Smoke-Taint: Stability During Fermentation with Different Yeasts and in Finished Wine

**Brandon Whitmore[1,●], Stephanie McCann[1], Matthew Noestheden[2,▲], Eric G Dennis[2], Sarah M Lyons[1,♦], Daniel M Durall[1], and Wesley F Zandberg[2,*]**

[1] The University of British Columbia, Department of Biology, 1177 Research Road, Kelowna, BC, Canada
[2] The University of British Columbia, Department of Chemistry, 3247 University Way, Kelowna, BC, Canada
Current addresses:

▲ SCIEX, 71 Four Valley Dr., Concord, ON, Canada, L4K 4V8
● University of Calgary, Cumming School of Medicine, 3330 Hospital Dr. NW, Calgary, AB, Canada, T2N 4N1
♦ Supra Research and Development, 2293 Leckie Road, Kelowna, BC, Canada, V1X 6Y5
* Correspondence: wesley.zandberg@ubc.ca ; Tel.: +1-250-807-9821

**Supporting Information Table of Contents**

Figure S1 – Spontaneous hydrolysis experimental design.

Table S1 – Model wine composition.

Table S2 – Model VP-glycosides used to fortify ferments with different strains of *S. cerevisiae*.

Table S3 – Recovery of free VPs from commercial Cabernet franc wine (pH 3.4) fortified with VP-glycosides (200 ng/g) after 12 weeks of spontaneous hydrolysis.

Table S4 – Change in total soluble solids (°Bx) as a metric of fermentation progress (days).

Figure S2 – Comparison of the concentration (ng/g) of VPs released by different yeast strains after primary fermentation.

**Figure S1**. Spontaneous hydrolysis of model VP-glycosides in Cabernet Franc wine and model wine matrices. All air/argon samples ($n$ = 3) were aliquoted (2 mL) into 4 mL borosilicate glass vials with polyvinyl-faced pulp lined caps and analyzed at the start of the experiment ($t$ = 0 weeks), after three months ($t$ = 12 weeks). * includes three separate 200 ng / mL fortifications as follows: 1) all β-D-glucopyranosides; 2) guaiacyl-primeveroside; 3) guaiacyl-gentiobioside and syringyl-gentiobioside.

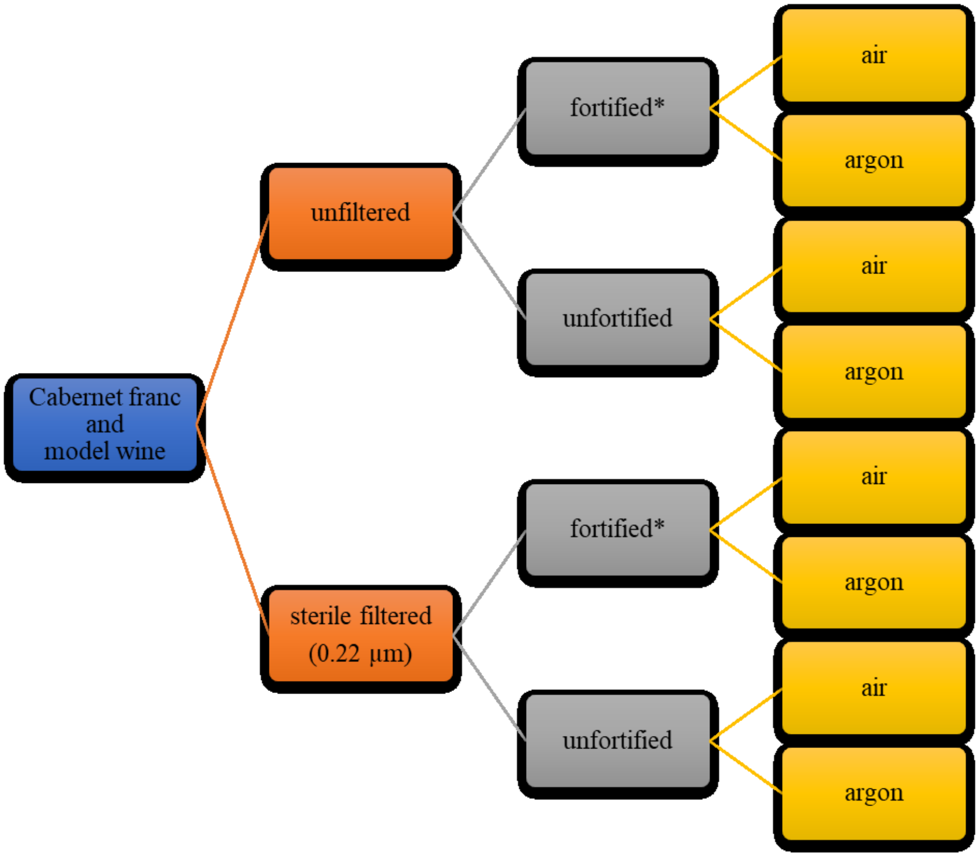

**Table S1**. Model wine composition. This mixture was used to validate the analysis of VPs in wine by GC-MS/MS, as it was free from incurred residues.

| **component** | **composition** |
|---|---|
| ethanol | 12 % (v/v) |
| tartaric acid | 5.0 g/L |
| malic acid | 3.5 g/L |
| acetic acid | 0.60 g/L |
| glucose | 2.0 g/L |
| fructose | 2.0 g/L |
| NaCl | 0.20 g/L |
| $KH_2PO_4$ | 2.0 g/L |
| $MgSO_4$ | 0.40 g/L |

**Table S2**. Model VP-glycosides used to fortify ferments with different strains of *S. cerevisiae*.

| compound | RT (min) | formula | exact mass (Da) |
|---|---|---|---|
| $d_3$-guaiacyl-β-D-glucopyranoside | 7.4 | $C_{13}H_{15}D_3O_7$ | 289.1241 |
| guaiacyl-β-D-glucopyranoside | 7.5 | $C_{13}H_{18}O_7$ | 286.1053 |
| guaiacyl-β-D-gentiobioside | 7.6 | $C_{19}H_{28}O_{12}$ | 448.1581 |
| *p*-cresyl-β-D-glucopyranoside | 8.8 | $C_{13}H_{18}O_6$ | 270.1103 |
| syringyl-β-D-gentiobioside | 9.1 | $C_{20}H_{30}O_{13}$ | 478.1686 |
| guaiacyl-β-D-primerveroside | 9.7 | $C_{18}H_{26}O_{11}$ | 418.1475 |
| syringyl-β-D-glucopyranoside | 10.1 | $C_{14}H_{20}O_8$ | 316.1158 |
| 4-methylguaiacyl-β-D-glucopyranoside | 11.4 | $C_{14}H_{20}O_7$ | 300.1209 |
| 4-ethylphenyl-β-D-glucopyranoside | 12.3 | $C_{13}H_{18}O_6$ | 270.1103 |
| eugenyl-β-D-glucopyranoside | 13.1 | $C_{16}H_{22}O_7$ | 326.1366 |

**Table S3**. Recovery of free VPs from commercial Cabernet franc wine (pH 3.4) fortified with VP-glycosides (200 ng/g) after 12 weeks of spontaneous hydrolysis.

| VP-glycoside | treatment | | concentration (ng/ g)[(1,2,3)] | | | | | | | |
|---|---|---|---|---|---|---|---|---|---|---|
| | **atmosphere** | **filtered** | **4-MG[(4)]** | **Gua** | **Phe** | ***p*-Cre** | ***o*-Cre** | **4-EP** | **Syr** | **Eug** |
| none | air | N | 1.1 ± 0.05 | 2.0 ± 0.20 | - | - | - | - | 1.6 ± 0.31 | 5.5 ± 0.26 |
| | | | 1.9 ± 0.25 | 4.1 ± 0.87 | 2.1 | 1.6 ± 0.29 | 2.1 ± 0.10 | 1.3 ± 0.16 | - | 10.0 ± 1.23 |
| | air | Y | 1.4 | **2.6 ± 0.18** | 3.2 ± 0.78 | 1.2 ± 0.10 | 2.6 ± 0.20 | **1.1 ± 0.01** | - | **7.2 ± 0.19** |
| | | | 1.6 ± 0.06 | **5.9 ± 0.32** | 1.7 ± 0.42 | 1.4 ± 0.12 | 1.8 ± 0.39 | **1.7 ± 0.14** | 6.3 ± 0.70 | **8.0 ± 0.15** |
| | argon | N | 1.2 | 5.8 ± 1.66 | - | **1.8 ± 0.05** | - | - | **65.9 ± 19.53** | 6.2 ± 0.25 |
| | | | 1.2 ± 0.03 | 9.4 ± 0.43 | 2.6 ± 0.22 | **2.17 ± 0.07** | - | 3.1 ± 0.22 | **159 ± 20** | 7.8 ± 0.58 |
| | argon | Y | 1.0 | 3.8 ± 0.16 | 1.7 ± 0.05 | 1.9 ± 0.17 | - | - | 43.8 ± 3.36 | 6.0 ± 0.47 |
| | | | - | 5.6 ± 1.96 | 2.2 ± 0.26 | 1.3 ± 0.06 | - | - | 94.5 ± 30.21 | 4.5 ± 0.22 |
| 4-MG-Glc<br>Gua-Glc<br>Phe-Glc<br>*p*-Cre-Glc<br>*o*-Cre-Glc<br>4-EP-Glc<br>Sry-Glc<br>Eug-Glc | air | N | 1.8 ± 0.85 | 6.0 ± 0.52 | - | **5.0 ± 0.28** | 1.7 ± 0.01 | **15.8 ± 0.85** | **2.7 ± 0.37** | 15.1 ± 0.54 |
| | | | 1.7 ± 0.04 | 6.8 ± 0.00 | - | **28.7 ± 0.09** | 1.2 ± 0.11 | **33.0 ± 0.43** | **6.7 ± 0.05** | 16.0 ± 0.53 |
| | air | Y | **1.5 ± 0.03** | **5.5 ± 0.43** | - | **4.3 ± 0.14** | **-** | **13.3 ± 0.08** | **2.4 ± 0.51** | **12.6 ± 0.30** |
| | | | **2.5 ± 0.12** | **11.3 ± 0.69** | 2.9 ± 0.60 | **55.7 ± 5.79** | **1.0 ± 0.00** | **56.5 ± 4.38** | **14.7 ± 1.53** | **24.0 ± 1.13** |
| | argon | N | **1.3 ± 0.06** | **11.1 ± 0.31** | **3.3 ± 0.19** | **5.0 ± 0.23** | - | **16.7 ± 0.52** | **129.1 ± 3.84** | **11.2 ± 0.85** |
| | | | **1.6 ± 0.04** | **12.3 ± 0.20** | **2.4 ± 0.20** | **38.2 ± 1.84** | - | **42.3 ± 1.70** | **186 ± 7.3** | **17.3 ± 1.13** |
| | argon | Y | 1.1 ± 0.04 | **8.2 ± 0.45** | 1.8 ± 0.34 | 5.0 ± 0.34 | - | 15.8 ± 0.59 | **63.3 ± 5.11** | 8.6 ± 0.41 |
| | | | 1.7 ± 0.22 | **14.8 ± 1.22** | 3.8 ± 0.96 | 44.6 ± 10.6 | 1.2 ± 0.09 | 49.9 ± 9.41 | **215 ± 5.6** | 21.4 ± 2.90 |
| Gua-Gent | air | N | **1.4 ± 0.05** | 3.7 ±0.31 | - | 1.2 | 1.5 ± 0.03 | 1.0 ± 0.02 | - | **7.3 ± 0.18** |
| | | | **1.1 ± 0.06** | 3.8 ± 0.38 | - | - | 1.3 ± 0.07 | - | 3.3 ± 0.58 | **6.1 ± 0.30** |
| | air | Y | 1.4 ± 0.04 | 3.6 ± 0.37 | - | 1.3 ± 0.10 | 2.1 ± 0.02 | 1.0 ± 0.01 | - | 6.9 ± 0.27 |
| | | | 1.3 ± 0.13 | 6.6 ± 1.23 | - | 1.4 ± 0.15 | 1.5 ± 0.38 | 1.8 ± 0.15 | 8.8 ± 2.35 | 6.9 ± 0.74 |
| Gua-Prim | air | N | **1.5 ± 0.09** | 4.4 ± 0.54 | - | 1.3 ± 0.10 | 1.6 | 1.2 ± 0.10 | - | **7.7 ± 0.42** |
| | | | **1.2 ± 0.06** | 3.7 ± 0.27 | - | - | - | 1.1 ± 0.07 | 4.1 ± 0.42 | **6.1 ± 0.20** |
| | air | Y | 1.1 ± 0.03 | **2.9 ± 0.19** | - | - | - | - | - | 5.7 ± 0.15 |
| | | | 1.4 ± 0.14 | **5.8 ± 0.66** | - | 1.2 ± 0.15 | 1.5 ± 0.27 | 1.4 ± 0.09 | 7.4 ± 0.56 | 6.7 ± 0.55 |
| Syr-Gent | air | N | 1.5 ± 0.07 | 4.6 ±0.19 | - | 1.3 | **1.6 ± 0.08** | 1.5 ± 0.16 | 1.6 | 8.5 ± 0.37 |
| | | | 1.2 ± 0.04 | 3.9 ± 0.62 | - | 1.1 ± 0.01 | **1.4 ± 0.09** | 1.5 ± 0.07 | 4.1 ± 0.80 | 6.7 ± 0.62 |
| | air | Y | 1.2 ± 0.16 | 4.9 ± 0.67 | 1.1 | 1.3 ± 0.08 | 1.1 | 1.6 ± 0.30 | **1.6 ± 0.40** | 7.7 ± 0.98 |
| | | | 1.4 ± 0.22 | 5.5 ± 0.96 | 1.8 | 1.3 ± 0.17 | - | 1.7 ± 0.27 | **8.0 ± 0.94** | 7.7 ± 0.78 |

**Notes:** (1) Fortified Cabernet franc wines were stored under an atmosphere of air or argon gas. When indicated, samples were filtered (Y) through a 0.22 μm filter prior to storage; untreated samples are denoted with a (N). (2) Data are provided as mean VP concentrations (in ng/g) recovered from each sample ± the standard error of the mean (SEM). For each sample, volatile phenol levels were determined after zero (top, shaded cells) or 12 weeks (bottom, unshaded cells) of storage. (3) Pairs of values in bold represent a statistically significant difference (two-tailed Welch's test of unequal variance, $n$ = 3/condition, $\alpha$ = 0.05) between samples analyzed at the two different time points for each condition. (4) The following abbreviations are used: Glc = β-D-glucopyranoside, Gent = gentiobioside, and Prim = primeveroside; 4-EP = 4-ethylphenol, 4-MG = 4-methylguaiacol, Eug = eugenol, Gua = guaiacol, *o*-Cre = *o*-cresol, *p*-Cre = *p*-cresol, Phe = phenol, and Syr = syringol.

**Table S4**. Change in total soluble solids (°Bx) as a metric of fermentation progress (days).

| strain | treatment | control | | | | | | smoked | | | | | |
|---|---|---|---|---|---|---|---|---|---|---|---|---|---|
| | fortified | | yes | | | no | | | yes | | | no | |
| | replicate | 1 | 2 | 3 | 1 | 2 | 3 | 1 | 2 | 3 | 1 | 2 | 3 |
| EC1118 | 0 | 21.7 | 22.2 | 22.1 | 21.9 | 21.3 | 22.2 | 22.7 | 23.1 | 23.2 | 22.9 | 23.4 | 21.7 |
| | 1 | 22.9 | 22.2 | 22.0 | 21.9 | 21.6 | 22.4 | 22.7 | 21.4 | 23.1 | 22.9 | 23.3 | 21.9 |
| | 2 | 20.7 | 18.5 | 21.4 | 19.2 | 18.7 | 19.4 | 17.6 | 16.6 | 18.9 | 19.3 | 20.7 | 18.9 |
| | 3 | 18.8 | 16.1 | 19.3 | 17.4 | 16.9 | 17.0 | 15.0 | 14.2 | 16.9 | 16.3 | 17.5 | 15.9 |
| | 4 | 14.5 | 13.1 | 16.1 | 13.7 | 13.4 | 13.7 | 11.3 | 10.8 | 12.3 | 12.6 | 14.4 | 12.2 |
| | 5 | 10.4 | 9.0 | 10.9 | 9.9 | 6.4 | 9.8 | 7.1 | 7.6 | 7.6 | 8.1 | 8.5 | 5.8 |
| | 6 | 6.8 | 5.9 | 6.5 | 7.8 | 5.9 | 7.1 | 6.7 | 6.2 | 7.4 | 7.4 | 8.2 | 7.1 |
| | 7 | 5.7 | 6.6 | 5.6 | 7.2 | 6.8 | 6.5 | 6.2 | 7.4 | 4.9 | 7.3 | 6.1 | 6.5 |
| | 8 | 6.9 | 7.9 | 6.0 | 6.1 | 5.5 | 6.9 | 5.9 | 6.3 | 6.2 | 5.8 | 6.5 | 5.9 |
| | 9 | 4.8 | 5.4 | 7.0 | 6.7 | 6.5 | 6.6 | 6.9 | 6.8 | 5.0 | 6.7 | 4.9 | 7.1 |
| RC212 | 0 | 21.4 | 21.2 | 21.5 | 20.2 | 20.4 | 21.7 | 17.4 | 18.3 | 19.4 | 18.4 | 18.4 | 18.0 |
| | 1 | 16.2 | 15.4 | 16.6 | 17.7 | 16.1 | 17.0 | 12.2 | 16.1 | 15.3 | 12.7 | 14.3 | 16.0 |
| | 2 | 12.1 | 13.2 | 14.3 | 13.4 | 14.8 | 13.7 | 10.1 | 10.2 | 11.0 | 12.4 | 13.6 | 10.6 |
| | 3 | 9.2 | 11.4 | 10.0 | 7.9 | 6.2 | 9.9 | 9.0 | 8.0 | 7.1 | 8.0 | 7.3 | 6.7 |
| | 4 | 8.9 | 8.6 | 6.6 | 8.5 | 7.9 | 8.6 | 6.2 | 6.8 | 5.5 | 6.6 | 5.9 | 5.8 |
| | 5 | 7.3 | 6.5 | 5.4 | 7.2 | 6.2 | 7.2 | 5.5 | 5.7 | 5.3 | 4.8 | 5.8 | 5.5 |
| | 6 | 5.3 | 5.5 | 6.7 | 6.2 | 5.3 | 6.0 | 5.2 | 5.3 | 5.6 | 5.6 | 5.2 | 5.2 |
| | 7 | - | 6.0 | 5.1 | 5.7 | 5.2 | 6.0 | 4.2 | 3.3 | 5.8 | 5.0 | 3.9 | 5.9 |
| BM45 | 0 | 20.4 | 22.0 | 19.1 | 20.3 | 21.4 | 20.6 | 19.6 | 19.2 | 20.1 | 19.4 | 19.7 | 19.8 |
| | 1 | 17.6 | 17.3 | 17.1 | 17.0 | 17.2 | 16.0 | 17.2 | 16.0 | 18.2 | 16.4 | 14.9 | 18.5 |
| | 2 | 10.4 | 9.2 | 11.4 | 11.9 | 11.4 | 12.6 | 15.7 | 11.3 | 13.0 | 12.3 | 10.8 | - |
| | 3 | 8.5 | 7.4 | 7.7 | 9.3 | 8.3 | 8.4 | 9.6 | 9.8 | 8.3 | 9.9 | 9.2 | 9.9 |
| | 4 | 7.3 | 7.0 | 5.9 | 6.3 | 6.9 | 5.8 | 9.4 | 9.3 | 9.7 | 8.4 | 9.6 | 9.8 |
| | 5 | 5.8 | 6.2 | 6.0 | 5.9 | 5.5 | 6.0 | 6.8 | 7.0 | 6.8 | 6.3 | 8.4 | 6.7 |
| | 6 | 6.3 | 7.1 | 6.0 | 6.0 | 6.4 | 5.6 | 6.4 | 5.7 | 5.3 | 6.2 | 6.7 | 6.1 |
| | 7 | 5.4 | 3.3 | 5.9 | 4.5 | 7.0 | 5.5 | 6.2 | 5.2 | 6.3 | 5.3 | 5.2 | 5.4 |
| U43 | 0 | | | | | | | | | | | | |
| | 1 | | | | | | | | | | | | |
| | 2 | 18.1 | 18.7 | 17.4 | 18.4 | 18.2 | 16.9 | 21.6 | 23.4 | 22.7 | 23.3 | 21.6 | 19.4 |
| | 3 | 16.1 | 20.7 | 17.7 | 17.2 | 15.4 | 14.9 | 19.5 | 18.7 | 18.5 | 22.6 | 17.5 | 17.5 |
| | 4 | 11.7 | 14.9 | 9.3 | 13.1 | 13.2 | 10.6 | 14.2 | 14.6 | 12.9 | 13.8 | 13.1 | 11.3 |
| | 5 | 8.2 | 6.4 | 8.9 | 9.0 | 9.2 | 6.8 | 6.7 | 8.4 | 9.0 | 6.8 | 9.7 | 6.6 |
| | 6 | 5.2 | 6.5 | 6.0 | 7.3 | 7.8 | 4.9 | 7.9 | 8.0 | 7.9 | 5.1 | 6.2 | 5.8 |
| | 7 | 4.4 | 6.4 | 3.7 | 4.0 | 5.9 | 5.9 | 2.5 | 6.0 | 5.7 | 4.3 | 6.7 | 5.4 |
| | 8 | 2.7 | 7.0 | 3.6 | 5.4 | 6.3 | 3.7 | 5.5 | 6.7 | 6.8 | 5.3 | 6.3 | 4.3 |

| strain | treatment | control | | | | | | smoked | | | | | |
|---|---|---|---|---|---|---|---|---|---|---|---|---|---|
| | fortified | yes | | | no | | | yes | | | no | | |
| | replicate | 1 | 2 | 3 | 1 | 2 | 3 | 1 | 2 | 3 | 1 | 2 | 3 |
| ES488 | 0 | | | | | | | | | | | | |
| | 1 | | | | | | | | | | | | |
| | 2 | 17.1 | 17.4 | 16.3 | 17.6 | 17.6 | 17.7 | 19.2 | 18.3 | 20.4 | 19.7 | 19.4 | 20.6 |
| | 3 | 16.7 | 17.9 | 16.5 | 14.8 | 16.5 | 19.2 | 19.7 | 17.7 | 20.4 | 18.3 | 19.1 | 21.2 |
| | 4 | 13.0 | 15.7 | 13.9 | 17.5 | 18.2 | 12.3 | 20.1 | 17.2 | 18.0 | 13.3 | 18.1 | 17.5 |
| | 5 | 10.0 | 13.2 | 11.3 | 14.7 | 11.2 | 9.7 | 15.4 | 9.5 | 11.4 | 16.3 | 13.4 | 10.8 |
| | 6 | 10.0 | 13.0 | 12.4 | 10.2 | 11.7 | 11.2 | 10.7 | 7.9 | 11.9 | 13.0 | 13.6 | 10.4 |
| | 7 | 9.6 | 9.5 | 8.5 | 6.5 | 6.6 | 8.9 | 8.7 | 7.8 | 7.0 | 8.8 | 7.4 | 8.0 |
| | 8 | 4.1 | 4.8 | 7.8 | 9.8 | 8.0 | 7.0 | 4.9 | 6.2 | 7.2 | 6.2 | 6.2 | 3.8 |
| Cru | 0 | | | | | | | | | | | | |
| | 1 | | | | | | | | | | | | |
| | 2 | 17.1 | 16.2 | 15.5 | 16.1 | 17.4 | 16.1 | 18.2 | 19.8 | 19.6 | 19.4 | 19.4 | 18.7 |
| | 3 | 16.6 | 15.2 | 13.6 | 16.0 | 14.3 | 14.6 | 13.7 | 19.9 | 17.1 | 20.7 | 20.6 | 18.3 |
| | 4 | 13.3 | 10.2 | 6.7 | 15.6 | 15.6 | 7.9 | 10.8 | 9.0 | 9.6 | 11.4 | 8.1 | 15.4 |
| | 5 | 11.6 | 11.2 | 8.7 | 6.9 | 10.6 | 10.2 | 5.5 | 8.9 | 6.7 | 11.8 | 10.6 | 10.5 |
| | 6 | 10.4 | 7.6 | 4.1 | 9.1 | 10.6 | 7.2 | 4.4 | 4.9 | 6.7 | 5.8 | 9.7 | 5.9 |
| | 7 | 5.4 | 8.9 | 7.3 | 5.3 | 8.0 | 7.9 | 5.1 | 3.5 | 3.4 | 7.8 | 5.2 | 6.0 |
| | 8 | 3.1 | 3.3 | 8.1 | 7.9 | 7.4 | 4.0 | 5.8 | 3.0 | 7.1 | 5.9 | 6.0 | 4.3 |

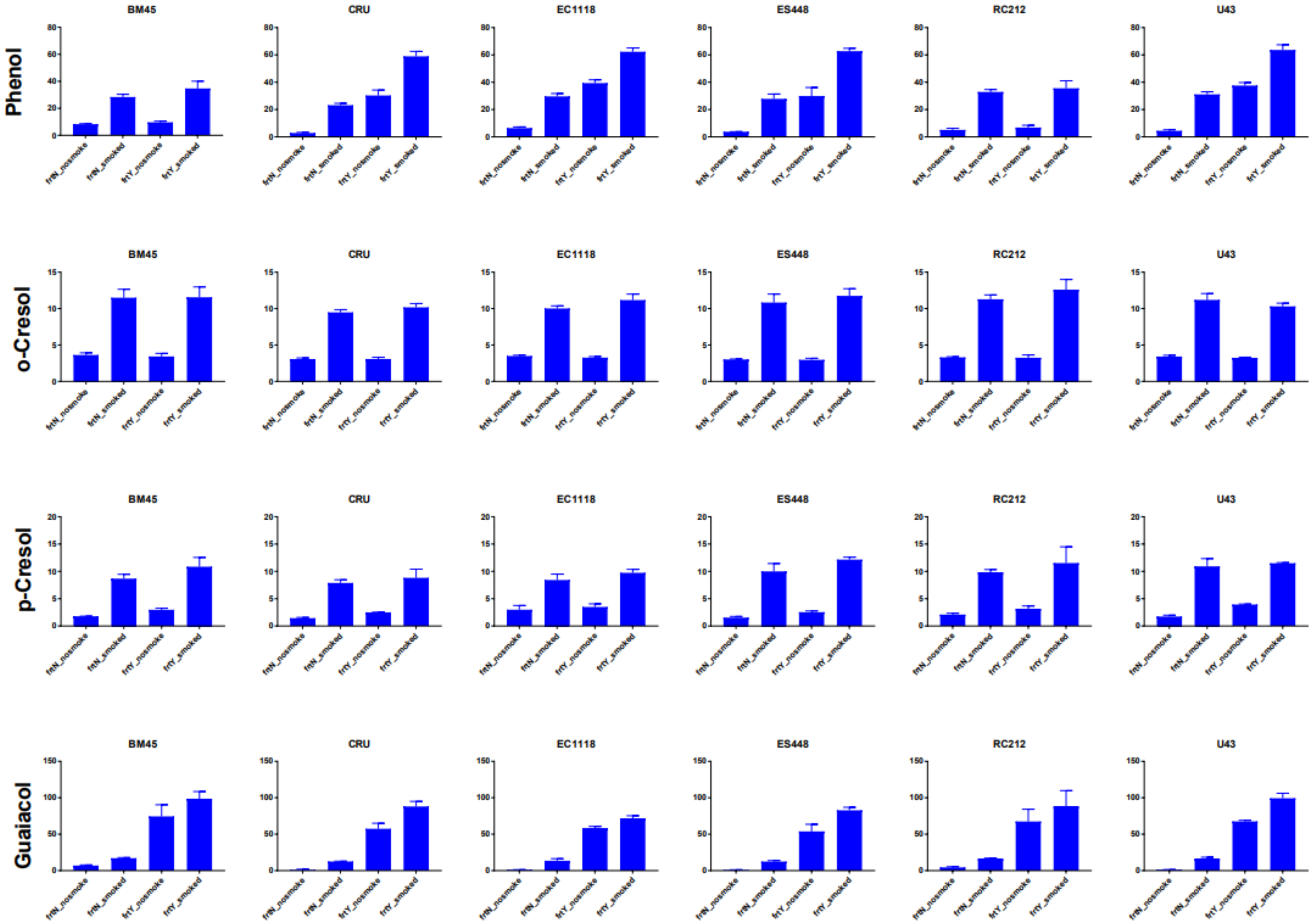
Phenol
o-Cresol
p-Cresol
Guaiacol
BM45
CRU
EC1118
ES448
RC212
U43
frtN_nosmoke
frtN_smoked
frtY_nosmoke
frtY_smoked

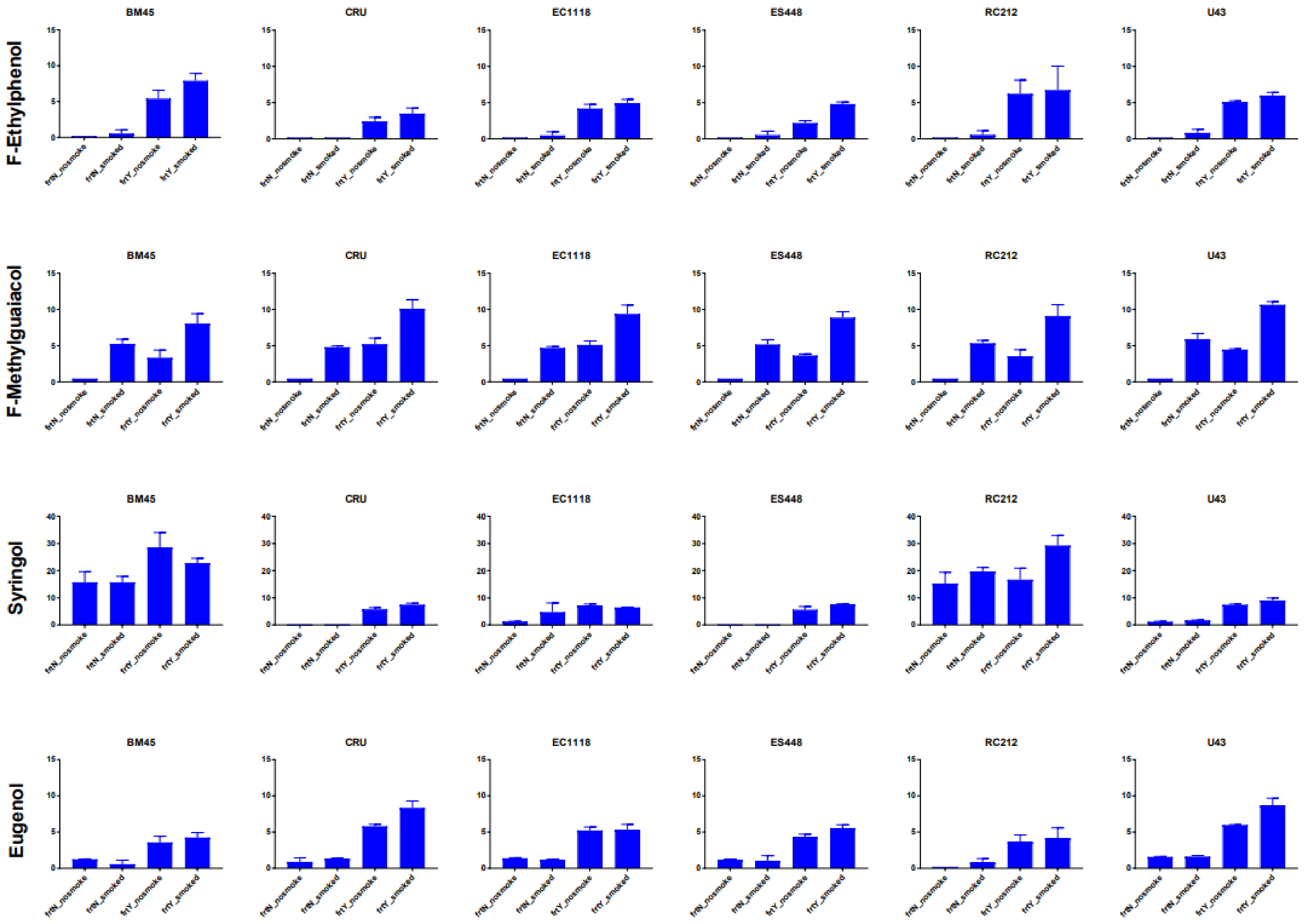
F-Ethylphenol
F-Methylguaiacol
Syringol
Eugenol
BM45
CRU
EC1118
ES448
RC212
U43
frtN_nosmoke
frtN_smoked
frtY_nosmoke
frtY_smoked

**Figure S2.** Comparison of the concentration (ng/g) of VPs released by different yeast strains after primary fermentation. Note that not every VP has the same *y*-axis scale. Error bars indicate the standard errors of the mean of $n = 3$ replicate fermentations. frtN and frtY indicate control or VP-glycoside-fortified samples, respectively, while "nosmoke" and "smoke" indicate Pinot Noir grapes were not or were exposed to simulated forest fire smoke while ripening.
